# Could the New Anti-CGRP Monoclonal Antibodies Be Effective in Migraine Aura? Case Reports and Literature Review

**DOI:** 10.3390/jcm11051228

**Published:** 2022-02-24

**Authors:** Maria Albanese, Nicola Biagio Mercuri

**Affiliations:** 1 Regional Referral Headache Center, Neurology Unit, University Hospital Tor Vergata, 00133 Rome, Italy; mercurin@med.uniroma2.it; 2 Department of Systems Medicine, University of Rome Tor Vergata, 00133 Rome, Italy; 3 IRCCS Santa Lucia Foundation, 00179 Rome, Italy

**Keywords:** migraine, headache, calcitonin gene-related peptide, monoclonal antibodies, migraine aura, cortical spreading depression, pain, triptans

## Abstract

Recently, monoclonal antibodies (mAbs) directed against calcitonin gene-related peptide (CGRP) (Eptinezumab, Fremanezumab, and Galcanezumab) or its receptor (Erenumab) have been approved for clinical use as prophylactic drugs for high-frequency episodic and chronic migraine. While their therapeutic effects on headache pain is well documented, there is scarce information on the usefulness of these medications in preventing migraine aura, which is believed to be associated with cortical spreading depression (CSD). Because of their large size, mAbs cannot easily cross the blood–brain barrier in high quantities, rendering the peripheral trigeminovascular system to likely be a major site of their action. In this paper, we report two cases of patients suffering from migraine with and without aura, who reported a complete disappearance of aura or reduced aura duration and intensity while taking Galcanezumab or Erenumab, respectively. Then, we present a brief overview of the literature about the controversial relationship between CSD and CGRP and about the potential “additional central” role of these mAbs in the pathophysiology of migraine aura.

## 1. Introduction

Migraine aura consists of focal reversible neurological deficits with a gradual and progressive onset that typically precedes or accompanies headache, or occurs without headache in up to one-third of patients. Clinical manifestations are extremely variable and may include alterations of sensitivity and disorders of language and/or of strength, but visual symptoms remain the most common. 

Many patients diagnosed with migraine aura (MA) occasionally have attacks of migraine without aura (MO) and vice-versa. Although the etiopathogenesis of MA has not yet been fully clarified, cortical spreading depression (CSD) seems to have a pivotal role and is closely connected to the release of calcitonin gene-related peptide (CGRP) [1,2]. In fact, CSD is a self-propagating wave of neuronal and glial depolarization that slowly spreads over the cortex, followed by a prolonged suppression of electrical activity. Apart from being the putative cause of the aura symptoms, CSD has been associated with neuroinflammation, probably contributing to the subsequent headache by activating the meningeal nociceptors and the central trigeminovascular neurons through the diffusion of substances released from the cortex (i.e., glutamate, potassium, H+, and ATP) [3,4,5]. In this mechanism, CGRP would be released from peripheral terminals in the pia and would help trigger neurogenic inflammation in the dura [6]. 

Several studies point towards important distinct familial, structural, and functional brain features between MA and MO [2,7,8]. MA may also respond differently to acute and prophylactic treatments as compared to MO [2,8]. 

Recently, new therapies have emerged for the management of episodic and chronic migraine in adults, including CGRP receptor antagonists (Gepants) and anti-CGRP monoclonal antibodies (mAbs) directed against CGRP (Eptinezumab, Fremanezumab, and Galcanezumab) or its receptor (Erenumab) [9,10]. Trials of the aforementioned drugs enrolled mixed populations of MA and MO, but the results were not stratified by the presence of aura. Moreover, mAbs have a larger molecule size compared to Gepants, so they cannot easily cross the blood–brain barrier (BBB) in high amounts and appear to accomplish their therapeutic effects by mainly targeting peripheral structures outside the central nervous system (CNS) [5,9,10].

According to the “neurovascular theory”, the activation of the trigeminovascular system (TGVS) and the release of numerous neuropeptides and vasoactive mediators (i.e., CGRP or substance P) play a key role in migraine pathogenesis [5]. Experimental data suggest that TGVS can also be activated by CSD, involving inflammatory cascades [4,5]. 

To date, although a known correlation exists between migraine, migraine aura, and CGRP release, the efficacy of these medications on the occurrence of auras and their possible effects on CSD remain to be elucidated. 

In relation to this issue, we report two cases of migraineurs who reported a complete disappearance of aura or reduced aura duration and intensity while taking Galcanezumab or Erenumab, respectively.

## 2. Case 1 Description

A 53-year-old female Caucasian patient of normal body weight (BMI 22 kg/m^2^) initially presented at our Headache Center of Tor Vergata in November 2020. She had suffered from migraines with and without aura since her childhood. She provided written informed consent to this discussion of her disease situation. 

The patient reported her headaches to be of high intensity (Numeric Rating Scale (NRS)—9/10), mostly unilateral (changing sides), with concomitant moderate photo- and phonophobia and a slight increase in headache during physical activity. 

**Figure 1:**
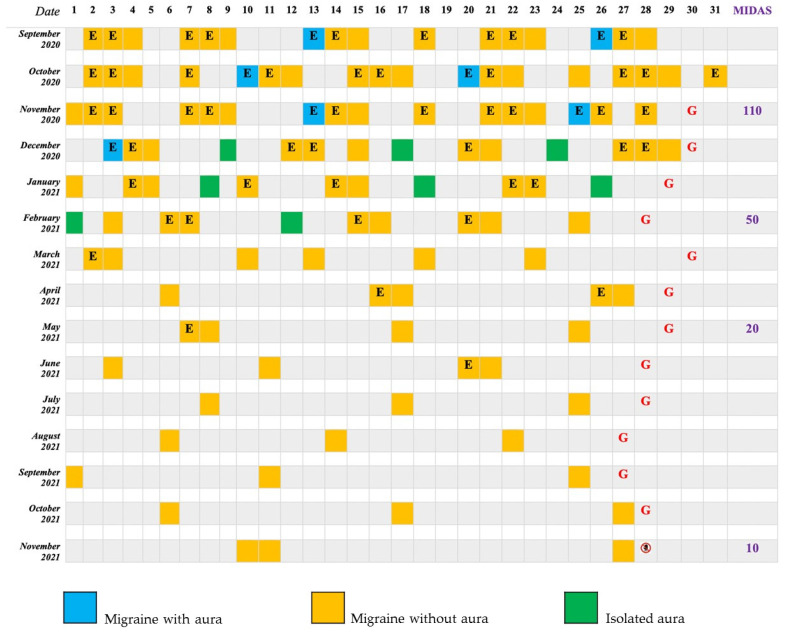
Patient 1 Migraine Diary. E: Eletriptan; G: Galcanezumab; MIDAS: Migraine Disability Assessment Test Score; 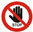 1 month after Galcanezumab discontinuation.

The attacks occurred 16–18 days per month and were preceded by visual auras twice per month, with severe resultant disability (Migraine Disability Assessment Test Score (MIDAS)—110 at presentation, see Figure 1). She described her auras presenting as “scintillating scotomas” associated with dimness that occurred in succession and evolved after 15–30 min into a headache.

Neurophthalmological examination performed during the visual phenomena was normal, not fulfilling the criteria for retinal migraine [11]. Moreover, all neuro-imaging studies and complementary tests were negative, performed to exclude other causes of transient blindness (i.e., vascular disorders). Family history of migraine and medical history were unremarkable. 

For acute therapy, the patient used oral Eletriptan (40 mg), with moderate success but with constant medication-overuse (more than 10 days per month). The patient was diagnosed with chronic migraine with and without aura and medication overuse, based on the International Classification of Headache Disorders 3rd edition (ICHD-3) [11]. Different prophylactic drugs were previously tried with sufficient dosing and time (anticonvulsants, antidepressants, beta-blockers, onabotulinumtoxinA, etc.), without beneficial effects. 

Therefore, Galcanezumab was administered, starting with a subcutaneous loading dosage of 240 mg and then 120 mg each month thereafter, according to the current Italian Medicines Agency (AIFA) migraine guidelines [12]. Over the following months, the patient reported a significant decrease of both frequency and intensity of migraine attacks (NRS 4/10), with only 3 migraine days at the end of treatment with Galcanezumab and a significant reduction in painkiller consumption as she did not need them as much anymore (see Figure 1).

Simultaneously, she noted an initial increase of aura episodes without headache, which completely disappeared 3 months after having begun the Galcanezumab treatment (see Figure 1). 

## 3. Case 2 Description

A 48-year-old man had been suffering from migraines with and without aura since he was 20 years old, but he did not have serious problems until last year, when headache episodes became more frequent and he had to refer many times to our Emergency Department. He provided written informed consent for this discussion of his medical case.

The attacks were characterized by unilateral beating pain, high intensity (NRS—9/10) together with nausea, vomiting, and photo-phonophobia. Visual migraine aura occurred every 15 days, with scotoma that spread gradually over ≥5 min and was followed by headache within 40 min.

Neurological examination and brain MRI without contrast were unremarkable. 

**Figure 2:**
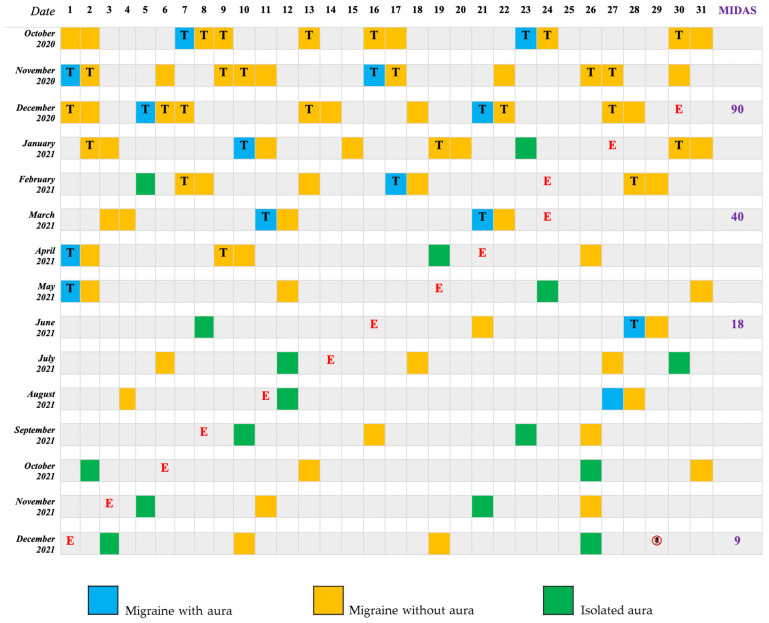
Patient 2 Migraine Diary. T: Triptans; E: Erenumab; MIDAS: Migraine Disability Assessment Test Score; 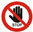 28 days after Erenumab discontinuation.

During his last presentation at our Emergency Room of Tor Vergata Hospital in December 2020, he reported a burden of 12 monthly migraine days, fulfilling the ICHD-III criteria for high-frequency episodic migraine [11], and rated 90 on MIDAS (see Figure 2). Several previous prophylactic drugs were reported to be useless (topiramate, lamotrigine, amitriptyline, and fluoxetine) or not tolerated (Beta-blockers due to sinusal bradicardya). Oral triptans were effective and often taken up to 3 doses/attack. Therefore, in line with the Italian migraine guidelines [12], a subcutaneous therapy was prescribed with the commercially available anti-CGRP receptor monoclonal antibody Erenumab at a dosage of 140 mg per month. 

After an 8-month treatment, migraine frequency decreased to 2 days per month and the use of triptans was gradually reduced (see Figure 2). However, visual auras continued to occur twice per month but with a shorter duration (15–20 min) and sometimes with a less intense pain (NRS—3/10) (see Figure 2).

## 4. Discussion

In these two cases of migraineurs, we showed that the new class of peripherally acting preventive mAbs could interfere with the clinical presentation of migraine aura. This may be due to indirect secondary changes after peripheral modulation of sensory input, or may represent an “additional central” mode of action. 

Although the etiopathogenesis of migraine aura is not fully clarified, evidence supports the hypothesis that Cortical Spreading Depression (CSD) is an underlying physiological process that could activate the trigeminal nociceptive system both peripherally and centrally in animal models, possibly provoking headache [13,14,15]. In fact, CSD is a self-propagating wave of neuronal and glial depolarization that slowly spreads over the cortex, fitting with the clinical picture of migraine aura [2,16,17]. Moreover, during the transient visual symptoms, fMRI studies suggest that BOLD signal changes develop in the occipital cortex and progress slowly, followed by local vascular perturbations mimicking CSD [2,18]. 

Despite the importance of both CSD and CGRP in migraine, the relationship between these two players remains unexplored and is controversial. There are several intriguing observations that indicate potential functional and anatomic connections between the two. 

First, in animal models, CGRP contributed to CSD-associated hypoperfusion and the secondary local changes due to CSD (i.e., elevated potassium or glutamate concentrations) caused a CGRP release at the cortical level [5]. The close correlation between CSD and CGRP release is also corroborated by the fact that endogenous CGRP was released during CSD in vitro cortical brain slices and in vivo CGRP receptor antagonism by Olcegepant significantly inhibited the occurrence of CSD [1].

A wide variety of experimental triggers, such as hypoxia or glyceryl trinitrate, provokes migraine attacks in susceptible subjects [19]. Moreover, CGRP infusion in patients with MA is able to trigger migraine-like attacks without aura and with the typical aura symptoms in 85% and 28% of cases, respectively [20].

Taken together, these data suggest that CGRP release following CSD events induces a positive feedback loop, facilitating the development of the CSD itself and an increased susceptibility to migraine. This may support the potential use and effectiveness of new therapies which target CGRP (Eptinezumab, Fremanezumab, and Galcanezumab) or its receptors (Erenumab) in order to prevent not only migraine pain but also migraine aura [13]. 

Until today, these molecules have demonstrated high responder rates and favorable adverse event profiles in the prophylaxis of both high-frequency episodic and chronic migraines, but studies on their efficacy in preventing migraine aura and CSD are still lacking [9,10]. Moreover, trials of these medications included mixed populations of adults, and the results were not stratified by the presence of aura [9,10].

A recent study using a murine model showed that CSD has effects on the spontaneous and evoked activity of high-threshold trigeminal neurons at spinal and dorsal horn levels. In the same study, the anti-CGRP-mAb Fremanezumab, administered intravenously in the rats, was able to prevent the CSD-mediated activation of central trigeminovascular neurons [21]. Fremanezumab and the other mAbs cannot easily cross the BBB due to their large size. Thus, it seems reasonable to hypothesize that they act mainly at peripheral sites and that their central effects are secondary to the inhibition of the activation of peripheral trigeminovascular neurons induced by CSD.

New data suggest that mAbs may have potential central properties, so they could influence CSD generation and the clinical manifestation of migraine aura, but the exact mechanisms remain unclear [22,23,24]. 

Since CGRP and its receptors are widely distributed throughout the cortex, it is possible that sufficient quantities of mAbs can reach the central nervous system (CNS) and inhibit CSD [22]. 

Recently, an fMRI study demonstrated that administering Erenumab modulates the activation of specific cerebral areas after trigeminal nociceptive input, including the thalamus, the insular cortex, and the secondary somatosensory cortex. It is only in responders’ patients that it induces a significantly reduced activation of the hypothalamus, a relevant central structure of migraine attack generation and chronification [23]. Moreover, Cevoli et al. reported a case of a man affected by chronic migraine with aura who presented a dramatic drop of both migraine and aura frequency after the first injection of Erenumab 70 mg monthly, with persistent efficacy after 1 year [24]. However, lamotrigine was previously taken before starting Erenumab, so the potential additional functional effect of this medication could not be excluded. In contrast, we observed an unchangeable aura frequency, but with a shorter duration and intensity, in a patient suffering from episodic migraine with and without aura. It may be hypothesized that the mode of action of Erenumab differs between episodic and chronic migraineurs (having fewer attacks does not activate the hypothalamus), in the presence of medication overuse or not, such as between patients suffering from both MA and MO and patients with only MA or MO, or between sex, gender, and species. 

Although no data for Erenumab have been published yet, another animal study showed the presence of Galcanezumab in peripheral and central tissues within 24 h of subcutaneous injections, which persisted for over 168 h [25]. There was a low level of access to Galcanezumab into the hypothalamus (0.34%) compared to its concentrations in the trigeminal ganglion and dura mater (5.2% and 11%, respectively), which is consistent with literature values available for other IgG proteins [25]. We cannot categorically exclude that a small amount of active medication in the hypothalamus might indeed be sufficient for a central effect and for reducing migraine aura as suggested in our case. Accordingly, here we propose that in our case, the delayed complete recovery of migraine aura corresponding to administration of Galcanezumab may be due to the capability of the drug to reach the CNS after a specific period, achieving a steady-state concentration (CSF/serum ratio) and saturable brain uptake similar to those of IgG proteins [25]. 

Nonetheless, previous studies using animal models of migraine aura found that even when the BBB was damaged and Fremanezumab was allowed to reach the cortex, it was able to slow down the propagation velocity of CSD and reduce the following cortical recovery period, but could not abolish the initiation of CSD depolarization waves [26]. Therefore, it may be possible that mAbs cannot completely block CSD because CSD properties are not exclusively CGRP-dependent. 

## 5. Conclusions

On this basis, real-world data and prospective studies with a higher level of standardization and larger sample sizes would be beneficial to clarify if these new drugs are able to modify the presentation of aura in patients suffering from such high-frequency forms of attacks or in patients suffering from both MA and MO. For patients, aura symptoms are alarming and may be transiently debilitating. A better understanding of the relationship between CSD, migraine aura, and CGRP and of the differential responses to therapy may be an important step towards more personalized medicine.

## Data Availability

Data is contained within the article.
